# Explained predictions of strong eastern Pacific El Niño events using deep learning

**DOI:** 10.1038/s41598-023-45739-3

**Published:** 2023-11-30

**Authors:** Gerardo A. Rivera Tello, Ken Takahashi, Christina Karamperidou

**Affiliations:** 1 Instituto Geofísico del Perú, Lima, Peru; 2 Department of Atmospheric Sciences, School of Ocean and Earth Science and Technology, University of Hawai’i at Mānoa, Honolulu, HI USA

**Keywords:** Climate sciences, Ocean sciences

## Abstract

Global and regional impacts of El Niño-Southern Oscillation (ENSO) are sensitive to the details of the pattern of anomalous ocean warming and cooling, such as the contrasts between the eastern and central Pacific. However, skillful prediction of such ENSO diversity remains a challenge even a few months in advance. Here, we present an experimental forecast with a deep learning model (IGP-UHM AI model v1.0) for the *E* (eastern Pacific) and *C* (central Pacific) ENSO diversity indices, specialized on the onset of strong eastern Pacific El Niño events by including a classification output. We find that higher ENSO nonlinearity is associated with better skill, with potential implications for ENSO predictability in a warming climate. When initialized in May 2023, our model predicts the persistence of El Niño conditions in the eastern Pacific into 2024, but with decreasing strength, similar to 2015–2016 but much weaker than 1997–1998. In contrast to the more typical El Niño development in 1997 and 2015, in addition to the ongoing eastern Pacific warming, an eXplainable Artificial Intelligence analysis for 2023 identifies weak warm surface, increased sea level and westerly wind anomalies in the western Pacific as precursors, countered by warm surface and southerly wind anomalies in the northern Atlantic.

## Introduction

El Niño-Southern Oscillation (ENSO) is the main source of interannual climate variability and predictability at the global scale due to its relatively slow ocean-atmosphere dynamics and fast atmospheric teleconnections. Extreme El Niño events, such as those in 1877–1878, 1982–1983 and 1997–1998, are characterized by very strong sea surface temperature (SST) anomalies and heavy precipitation associated with deep atmospheric convection in the otherwise stable eastern Pacific. Their abnormal growth relies on nonlinearly amplified atmospheric feedbacks, associated with this deep convection^1–4^. During such extreme basin-scale eastern Pacific El Niño events, as well as during extreme coastal El Niño events as in early 1925, 2017 and 2023^5–7^, heavy flooding continues to produce the largest socio-economic impacts along the coast^8–11^; it was these direct effects that led to the original identification of El Niño in the late 19th century^12,13^ as anomalously warm and rainy conditions along the coast of Peru.

Modern ENSO forecast systems are based on coupled ocean-atmosphere general circulation models (GCMs) that are initialized with observational data, and generally have good skill. For example, for February sea surface temperature (SST) anomalies in the Niño 3.4 region in the central equatorial Pacific (5$^{\circ }$N–5$^{\circ }$S, 170$^{\circ }$W–120$^{\circ }$W), a correlation of 0.7 can be achieved with a 9-month lead^14^. However, El Niño impacts are strongly sensitive to the SST anomaly pattern; for example, in Peru, which is a hotspot for ENSO direct impacts, eastern Pacific warming results in an increase in precipitation in its coastal region, while central Pacific warming leads to a decrease in precipitation in its Andean region^15^. Similarly, in Ecuador, central, eastern, and coastal El Niño events have significantly different impacts^16^. Unfortunately, the forecast skill for eastern and central Pacific ENSO events, dubbed ENSO diversity^17^, is still very limited. ref^18^ showed that Eastern and central Pacific El Niño types could only be distinguished by up to 3 out of 6 models in predictions for December–February initialized in November. For extreme eastern Pacific El Niño, the strength of the warming is critical, yet independent verification of the operational forecasts during the 1997–1998 event indicated that the existing forecast models did not recognize its exceptional strength until June 1997^19^. While the strong 2015–2016 El Niño was not an extreme eastern Pacific event and resulted in dry conditions along the eastern Pacific coast, the operational dynamical models still underestimated its strength until July 2015, even for the Niño 3.4 region^20^. This could partially reflect that the intrinsic ENSO amplitudes vary among models and that their spatial biases, including long-standing common warm and wet coastal biases in the eastern Pacific and the cold equatorial Pacific bias^21^, imply zonal shifts of the Niño regions in each model. It has been shown that representing ENSO variability in GCMs using indices derived from their respective variability patterns, such as the *E* and *C* indices^2^, which are implicitly bias-corrected, can help identify those models that can adequately represent key aspects of ENSO dynamics, including nonlinearities^3,22,23^ and which can thus be considered more realistic for future climate projections and for seasonal prediction^24^. To wit, half or more of the CMIP5 and 6 models do not adequately simulate the degree of ENSO nonlinearity and diversity^22,25^, which casts doubt on their ability to be used for skillful predictions of the strength and pattern of warming during El Niño, especially in the far eastern Pacific^26^. Although multi-model ensembles help with the cancellation of errors and the estimation of uncertainty^27^, GCMs and their initialization systems are not independent^27,28^, so a strong multi-model consensus is not necessarily an indication of low uncertainty, as illustrated by the consistent but ultimately failed multi-model forecasts of a substantial El Niño in 2014^29^.

In forecasting, it is preferable to use a well-calibrated model over subjective judgment if available^30^, but observational data for tuning models for strong El Niño prediction are limited, e.g., only the 1982–1983 and 1997–1998 extreme events have been comprehensively observed. Besides, GCMs are not explicitly tuned for prediction, although their output can be post-processed, so several operational ENSO forecasting centers rely on human judgment for the final product^20,31^. Even in the case of weather forecasts, in which feedback is much more frequent and models could theoretically be better calibrated, expert forecasters in principle remain skeptical and use their expertise with the models and their meteorological knowledge to assess and use the model results^32^. The World Meteorological Organization recommends offering physical explanations of a forecast to increase the confidence of the users^33^, but the complexity of GCMs is so high that they are essentially “black boxes”, i.e., we cannot directly and readily explain why a GCM makes a specific prediction despite knowing and understanding the underlying physical equations. The need for transparency has recently become more prominent in the context of artificial intelligence, particularly for high-stakes applications^34^, such as early warnings of climate hazards^35^. EXplainable Artificial Intelligence (XAI) techniques may provide much-needed real-time insights to human forecasters, by highlighting the observed climate variables and corresponding geographical regions most relevant for the predictions, both for and against them^36^, thus making AI models a valuable addition to the forecaster’s toolbox even in cases where they do not outperform the GCMs.

The application of AI models to ENSO prediction has been shown to overcome some of the limitations in GCMs, such as the predictability barrier, and can produce accurate long-range predictions^37–42^; these models can also be used to generate and test hypotheses to enhance our understanding of ENSO dynamics^43^. However, to date, less attention has been given to predicting ENSO diversity, especially to developing models that address the need for increasing the accuracy of predictions of ENSO diversity beyond a few months, particularly of strong eastern Pacific El Niño events, for which nonlinear processes are critical for the representation of El Niño development.

In current international forecasts, most models are calling for the ongoing El Niño to extend or even strengthen into the 2023–2024 austral summer/boreal winter. For instance, the official USA probabilistic forecast for El Niño in the Niño 3.4 region (issued June 8, 2023) is 96% for December 2023–February 2024, with 50% for a strong event^44^. On the other hand, the corresponding official probability forecasts from Peru (issued June 16, 2023) for December 2023–March 2024 are 88% and 6% for the Niño 3.4 region and 84% and 10% for the Niño 1+2, in the far-eastern Pacific, off the coast of Peru^45^. The substantial difference in the strong category between the two assessments despite using similar information, including the GCM forecasts, is a result of the fact that they are ultimately produced by human forecasters, as well as to somewhat different operational definitions^20,31^.

Given the possibility of a strong El Niño event in late 2023 and the need for trustworthy tools for generating and explaining the forecasts, we present in this study an El Niño forecast up to April 2024, initialized with observational data for March–May 2023, using a skillful convolutional neural network model (IGP-UHM AI v1.0). Our model predicts the *E* and *C* indices with up to 12-month lead, and is trained specifically for the prediction of strong eastern Pacific events, where the E index exceeds 1.5 standard deviations in austral summer^3^. The IGP-UHM AI model was trained initially using a total of more than 3200 years from the “historical” simulations of 21 CMIP6 GCMs, which were selected according to their ability to simulate the nonlinear relationship between the *E* and *C* indices^22^. Although the main interest is in the prediction of the onset of extreme events (approximately $E>3$), since by definition these are very rare, we focused the training by oversampling the data for strong eastern Pacific events ($E>1.5$) and by adding a classification output, so the model also predicts the probability of strong events in the following January. We then fine-tuned the model with observational data for the 1871–1984 period and tested it with data for the 1990–2022 period, as well as generated a forecast for June 2023–May 2024. Using an eXplainable Artificial Intelligence method, we analyze the explanations for the strong event predictions, focusing on the 1997–1998 and 2015–2016 events and comparing them to the current forecast. Unlike previous work^39–42,46^, the focus of our work is primarily geared towards predicting ENSO diversity, with a special emphasis on predicting strong Eastern Pacific events via a skillful model designed to be eventually used in an operational setting, which therefore includes an explanatory framework that is consistent with our understanding of the physical processes governing ENSO diversity. In particular, our model uses four real-time observable input variables that are important for a critical evaluation of the forecasts (SST, sea surface height, and zonal and meridional winds), as opposed to only using SST and heat content that is not directly observed^37–39,41,42,46^.

## Results

### IGP-UHM AI model performance

**Figure 1:**
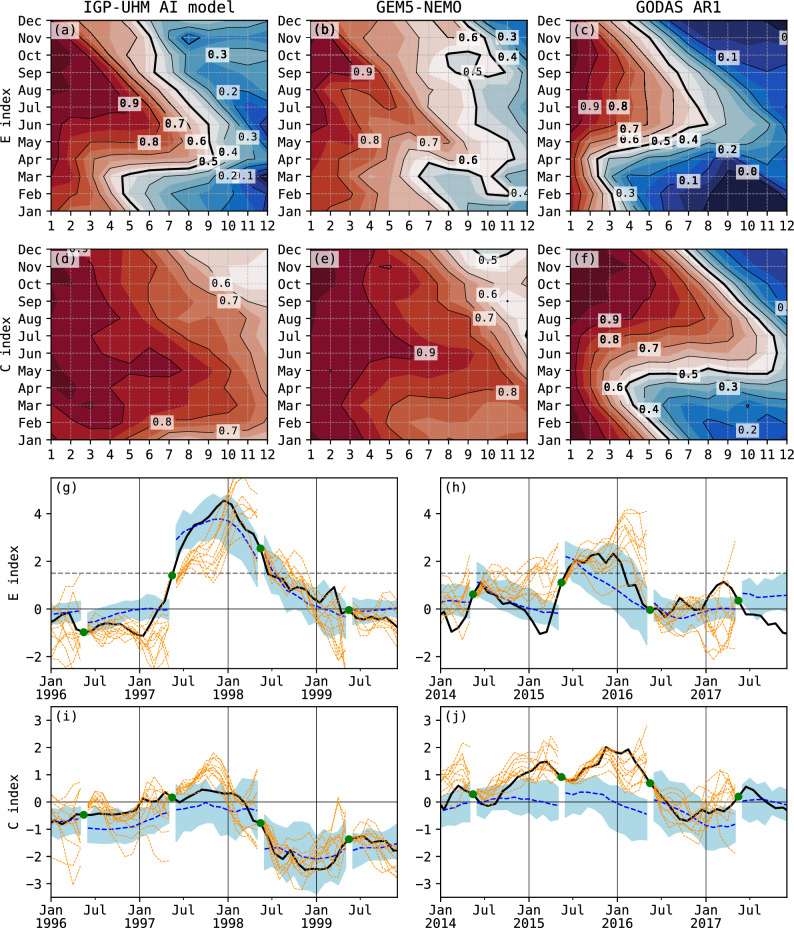
Performance of the IGP-UHM AI model. Top panels: Correlation coefficient between the observed and predicted (**a**–**c**) ensemble-mean *E* and (**d**–**f**) *C* indices as a function of lead time (months) and initial condition for the (**a**,**d**) IGP-UHM AI model, (**b**,**e**) the GEM5-NEMO model, and (**c**,**f**) an AR1 statistical model. Bottom panels: Observed (black) and predicted values with May initialization for (**g**,**h**) *E* and (**i**,**j**) *C* indices for the periods (**g**,**i**) 1996–1999 and (**h**,**j**) 2014–2017. The IGP-UHM AI ensemble mean is shown in the blue dashed line and the ensemble spread in light blue shading. The GEM5-NEMO ensemble members are shown in orange. Green dots indicate May conditions.

The IGP-UHM AI model produces skillful predictions for the *E* and *C* indices, as shown by the correlation coefficients between the observed and predicted values of the indices for the independent testing dataset, stratified by the month of the initial conditions and the lead time of prediction (Fig. 1). The correlations for *E* of the IGP-UHM AI model (Fig. 1a) are comparable to those of the GEM5-NEMO model^47^ (Fig. 1b), which is a particularly skillful GCM for the long-range prediction of *E*^24^. If we consider a correlation coefficient target of 0.7 or larger, the longest lead in both cases is around 8 months for May initial conditions. For May initial conditions and a lead time of 8 months, the IGP-UHM AI model performs well in predicting both the amplitude and the phase of the indices (Supplementary Fig. 1), with correlation coefficients 0.66 for the E and 0.89 for the C index over the entire observational testing period (1990–2022). Both the IGP-UHM AI and the GEM5-NEMO model show a seasonal predictability barrier for E in austral fall/boreal spring, with the shortest lead time of around 3 months with March initial conditions (Fig. 1a,b). The correlation coefficients for both models are substantially higher than those for an AR1 model, i.e., the index lagged autocorrelations, for which the longest leads are found later in the year, for June initial conditions (Fig. 1c). For the *C* index, the two models’ correlations (Fig. 1d,e) are generally higher than for *E* and without an apparent predictability barrier, which is very prominent in the *C* lagged autocorrelations (Fig. 1f).

A skill metric more relevant to strong El Niño events is needed, as the correlation coefficients combine El Niño and La Niña events, as well as weak, moderate and strong events. We thus calculate the Critical Success Index (CSI) for the classification problem of determining whether a strong event will occur in austral summer ($E>1.5$ in January). The CSI for May initial conditions for the 1990–2022 testing period is 0.67, which is the same as for the GEM5-NEMO model. For our model, this CSI value reflects two true positives, i.e., correct predictions of a strong El Niño in January with May 1997 and 2015 initial conditions, one false positive with May 2002 initial conditions, 30 true negatives, and 0 false negatives.

For the two true positive predictions of strong eastern Pacific El Niño events in 1997–1998 and 2015–2016, the IGP-UHM AI model closely predicts the onset and decay of *E*, while the peak is better predicted in 1997–1998 compared to 2015–2016. These two events were different in the relative strengths of *E* and *C*, with the 1997–1998 event classifying as extreme ($E>4$) in contrast to the strong 2015–2016 event ($E\approx 2$), which had comparatively weak warming in the eastern Pacific^20,48^ and has been classified as a “mixed” eastern and central Pacific event. Using May 1997 initial conditions, the IGP-UHM AI model performed very well in predicting the monthly evolution of the *E* index, particularly the onset and the peak values in austral summer. The observed and predicted E and C values for January 1998 were 4.3 and 0.31 vs 3.7 and − 0.29, respectively. The correlations between the observed and predicted values for the 1997 prediction were 0.85 for E and 0.47 for C, indicating a higher prediction skill for E in this strong case. The ensemble mean prediction for C, while negative, is still within the neutral range (− 0.5 to 0.5) as is the observed positive value (0.31). In fact, the model predicts a 67% probability for C within the neutral range. Hence, the IGP-UHM AI model adequately predicts the *C* evolution during the 1997-98 event, in contrast to its overestimation by the GEM5-NEMO model (Fig. 1i). The GEM5-NEMO model also predicted high values of *E*, although with a delayed onset and peak (Fig. 1g). For the 2015–2016 event, the E and C observed vs predicted values were 1.95 and 1.77 vs 0.77 and −0.04, respectively. The correlation for the E index was 0.75 while for the C index was 0.03, showing again the disparity in skill. Both the IGP-UHM AI and the GEM5-NEMO model predicted the onset adequately, but while the IGP-UHM AI model correctly predicted the decay during the first half of 2016, the GEM5-NEMO model generally predicted further warming (Fig. 1h). In contrast, the GEM5-NEMO model accurately predicted the 2015–2016 *C* evolution but the IGP-UHM AI model was unable to predict the central Pacific warming that was an important feature of this event (Fig. 1j). Thus, in summary, both models could differentiate the strength of the warming in the eastern Pacific in the two events, but the IGP-UHM AI model better predicted the evolution of *E*. We suspect that the lower skill in predicting the evolution of C originates in the training strategy that prioritized the eastern Pacific, but may also be due to the fact that central Pacific El Niño events appear to have doubled since 1980^49^ and the fine-tuning of the model did not fully expose it to these conditions.

**Figure 2:**
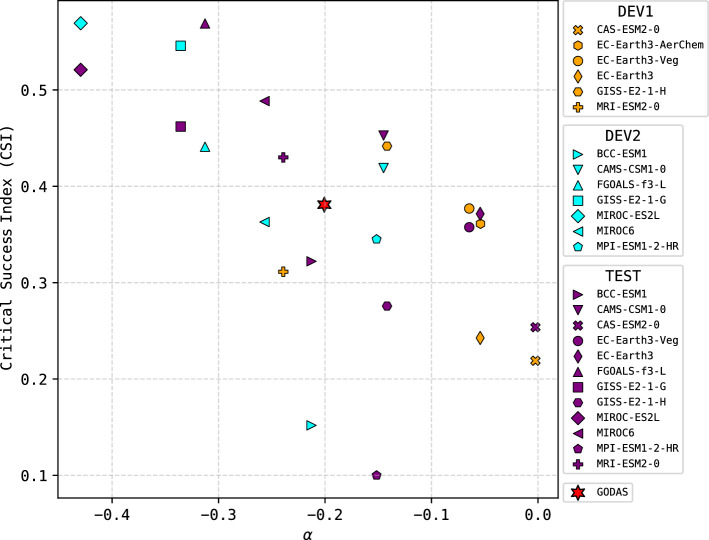
Scatter plot of the aggregated Critical Success Index (CSI) vs. the ENSO nonlinearity coefficient $\alpha$. The CSI is a skill metric for deep learning predictions of strong eastern Pacific El Niño events ($E>1.5$ in January) aggregated over the forecasts initialized in the preceding 12 months using different GCM data as input. The coefficient $\alpha$ is a metric for ENSO diversity calculated based on each model’s historical simulation. The red star indicates the observational estimate of $\alpha$ vs the IGP-UHM AI model’s CSI for the observed 1990–2022 period. Colors indicate the subsets of GCM data used in the two development stages (DEV1, DEV2) and the testing stage (TEST).

An interesting finding is that the CSI for the predictions corresponding to different CMIP6 GCMs, using our model without fine-tuning, shows a direct relationship to the degree of nonlinearity of ENSO in the GCMs, as measured by coefficient $\alpha$^22^ (Fig. 2). This suggests a causal relationship, i.e., models that have stronger ENSO nonlinearities produce strong eastern Pacific El Niño events that are more predictable. An alternative hypothesis is that this correlation emerges from a bias in the training, as GCMs with stronger nonlinearities could contribute with more El Niño events in the training dataset, i.e., better skill would be a result of better training of the CNN to those GCMs, but we discarded this hypothesis because considering both the nonlinearity and the number of events per GCM as linear predictors of CSI for the independent testing dataset yields nearly the same coefficient of determination as using the nonlinearity alone ($R^2 = 0.43$ and $R^2 = 0.41$, respectively). The results for the IGP-UHM AI model in the 1990–2022 period lie within the relationship found across the GCM results (star in Fig. 2), suggesting that the observed nonlinearity is a constraint on the skill level that can be achieved with the AI model, i.e., if the observed nonlinearity were higher we might expect higher predictability and prediction skill^50^.

### Explanations of the predictions of strong El Niño events

**Figure 3:**
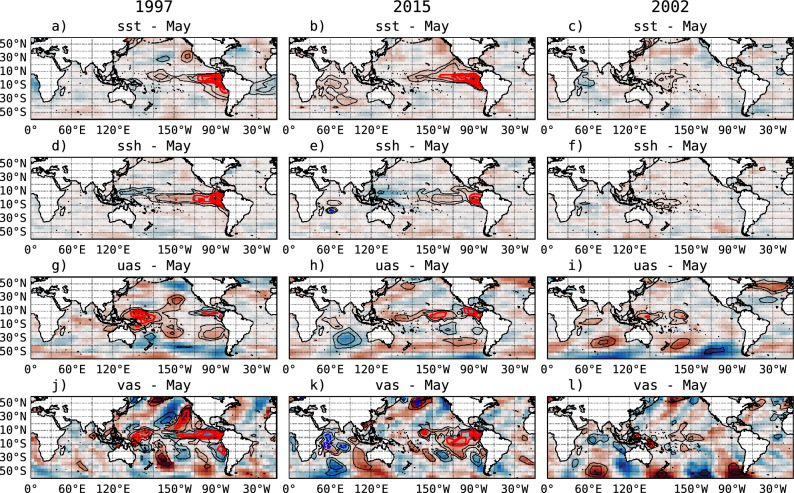
Standardized anomaly maps (shading) and Layerwise-Relevance-Propagation (LRP) values (contours) for the IGP-UHM AI model predictor fields: SST (**a**–**c**), SSH (**d**–**f**), zonal (**g**–**i**) and meridional wind (**j**–**l**), for May initial conditions (March and April not shown) for the years 1997 (**a**,**d**,**g**,**j**), 2015 (**b**,**e**,**h**,**k**) and 2002 (**c**,**f**,**i**,**l**). The anomaly values are scaled as detailed in the “Methods” section. The ensemble-mean LRP relevance for the classification prediction of $E>1.5$ in the following January is shown in contours, with the strongest positive and negative contributions shown in solid red and dashed blue, respectively.

Using XAI methods, we can determine the most relevant features in the input to the IGP-UHM AI model for a given strong EP El Niño probability from the classification output (Fig. 3). The Layerwise Relevance Propagation (LRP) technique produces a “relevance” value for each grid point in the 12 input fields (4 variables, 3 times) such that positive values indicate favorable conditions for the prediction of a strong eastern Pacific El Niño, whereas negative values are unfavorable. The relevance values are scaled so that the probability of a strong eastern Pacific El Niño is proportional to the exponential of their sum over all grid points of the corresponding 12 input fields.

The model predicted a 99.6% probability of a strong EP El Niño in January of 1998 using May 1997 initial conditions, so the LRP relevance fields are expected to be dominated by positive values. At first glance, the most favorable conditions (high relevance values) are as could be expected: high SST and sea surface height (SSH) in the eastern Pacific and westerly wind anomalies in the central-western equatorial Pacific^3^, the latter with a southerly component (Fig. 3a,d,g,j, respectively). Less well known perhaps is that northerly wind anomalies in the eastern equatorial (with an easterly component) and south-eastern Pacific are also considered important by the IGP-UHM AI model (Fig. 3j), as has been previously identified^51^. Also considered relevant are southerly wind anomalies in the north-eastern Pacific (around 130$^\circ$W, 10–40$^\circ$N, Fig. 3j), consistent with the wind signature of the positive phase of the Pacific Meridional Mode, which is favorable to the development of eastern Pacific El Niño events^52^.

The forecasted probability for January 2016 with May 2015 initial conditions was only 56.6%, much lower than for 1997. Thus, although the LRP maps have several similarities to 1997, particularly the focus on the positive SST and SSH anomalies in the eastern and westerly wind anomalies in the central-eastern region (around 140$^\circ$W) of the equatorial Pacific, the relevance values are smaller, particularly for the wind (Fig. 3b,e,h,k). Additionally, the northerly wind anomalies in the eastern equatorial and southeastern Pacific are weak or absent compared to 1997 (Fig. 3b,e,k). This difference in the meridional winds was noted by the ENFEN (Estudio Nacional del Fenómeno El Niño) Commission in Peru in August 2015 and was considered an unfavorable condition for the development of an extreme eastern Pacific El Niño event similar to 1997–1998^53^. On the other hand, while high latitudes show strong anomalies in both the southern and the northern hemisphere (Fig. 3j–l), these are not considered by the model for the prediction, which is indicated by the lack of contours in those regions.

The singular false positive prediction of strong EP El Niño was for January 2003 with a very high probability of 92.3%. This is an intriguing case, as no strong precursors of El Niño were present in the equatorial Pacific in May 2002 (Fig. 3c,f,i,l). Only weak westerly wind anomalies in the central and western region and southerly anomalies in the far western region are seen, as well as weak positive SST and SSH anomalies in the western-central region. Higher relevance is assigned to the wind anomalies, which were present to some extent in March and April (not shown here). Another weak feature deemed somewhat relevant according to LRP was an anomalous anticyclonic circulation in the north Atlantic (Fig. 3i,l), also present in March and April, consistent with the positive phase of the North Atlantic Oscillation (NAO). However, to our knowledge, no role has been found for NAO as a precursor to an El Niño event, which might indicate that the IGP-UHM AI is incorrectly considering this as relevant; as it also did in May 2015 with respect to the southerly signal in the north Pacific (Fig. 3k). This highlights the need for the LRP explanations to be analyzed by the forecasters to assess the IGP-UHM AI predictions if considered as guidance for operational predictions.

### Prediction for the 2023–2024 austral summer

**Figure 4:**
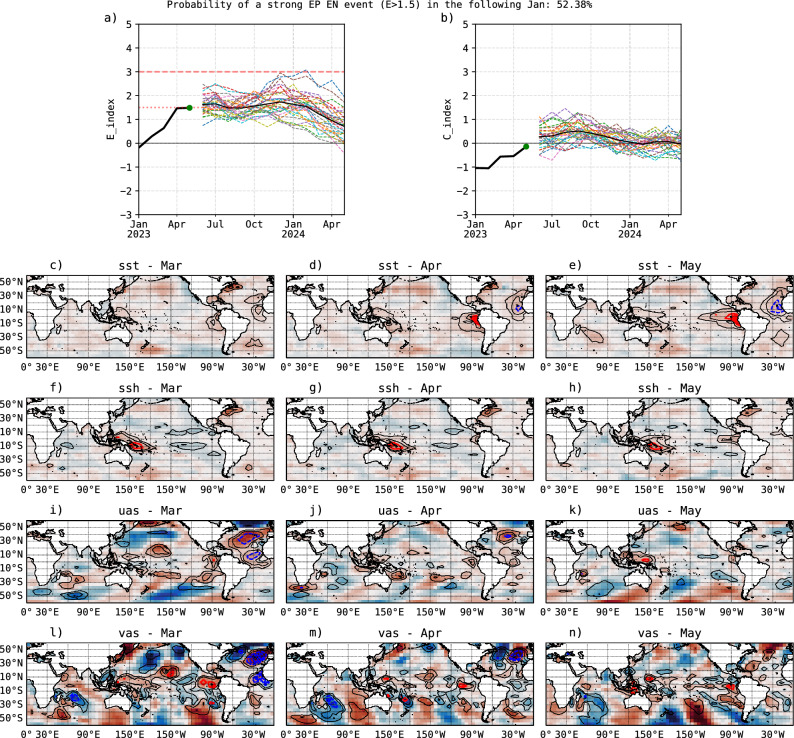
IGP-UHM AI model forecasts of (**a**) *E* and (**b**) *C* with May 2023 initial conditions, with observed values in black. (**c**–**n**) Predictor and LRP relevance maps as in Fig. 3, for (**c**,**f**,**i**,**l**) March, (**d**,**g**,**j**,**m**) April, and (**e**,**h**,**k**,**n**) May 2023. The LRP maps indicate the conditions that contribute positively (solid contours) and negatively (dashed contours) to the January 2024 prediction.

The ultimate test of a forecast model is to predict the future, as even subjective knowledge of the test data reserved as independent could influence the model development. The IGP-UHM AI prediction for *E* during 2023–2024 with May 2023 initial conditions is similar to the prediction for 2015–2016 discussed previously, with a 52.4% probability of a strong eastern Pacific El Niño and *E* decreasing progressively into austral summer, with an ensemble mean value for January of $E=1.63$, ranging between 0.5 and 2.8 (Fig. 4a). For *C*, the forecast is around neutral for the summer, with a range of $C=-0.5$ to 0.8 (Fig. 4b). This forecast is similar to the one with May 2015 initial conditions (Fig. 1h,j), yet the initial conditions and LRP explanations are quite different (Fig. 4c–n). For comparison, the IRI ensemble forecast with May initial conditions gave a 56% chance of November–January Niño-3.4 being above 1.5 °C with an 84% chance of exceeding moderate strength (Niño-3.4 $\ge$ 1.0 °C)^54^.

The most evident feature in the initial conditions is the warm SST anomalies in the far-eastern Pacific, associated with the ongoing “coastal El Niño”, similar to the ones in 1925 and 2017^5,6^. While the coastal event started in February 2023, its associated anomalies are identified as most relevant in April and May 2023 (Fig. 4d,e). The other most relevant feature is the positive SSH anomalies in the western equatorial Pacific (150–170$^\circ$E, 15-5$^\circ$S) in March–May 2023 (Fig. 4f–h). These two features are part of the patterns of the optimal initial structures for the development of an eastern Pacific El Niño in a linear model^52^, but one notable missing element is the positive SSH anomaly along the central and eastern equatorial Pacific that was present in 1997 (Fig. 3d).

Additionally, equatorial westerly wind anomalies are only seen in May 2023 and only in the far-western equatorial Pacific (120–150$^\circ$E; Fig. 4k), much less pronounced than in 1997 and even 2015. In this respect, recent operational assessments (June/July 2023) recognize that the atmosphere is not fully coupled to the ocean, a key element for El Niño-Southern Oscillation^55,56^, and is, therefore, a source of uncertainty for the further development of this El Niño event into the austral summer.

Furthermore, similarly to 2015, the northerly wind anomalies in the eastern equatorial and southeastern Pacific that were considered favorable for a strong eastern Pacific El Niño event in 1997 have not been present in 2023, except for a hint in the southeastern Pacific in March (Fig. 4l–n).

On the other hand, LRP shows unfavorable conditions, i.e., negative relevance, for a strong eastern Pacific El Niño associated with positive SST anomalies in the northeastern tropical Atlantic region, particularly in April and May (Fig. 4d,e). This is consistent with the documented negative effect of North Tropical Atlantic (NTA) warming and the Atlantic Niño on the central equatorial Pacific SST and zonal winds^57–59^, which in this case could be countering the atmospheric coupling associated with the Bjerknes feedback.

In summary, the model predictions for a slowly decaying *E* and a low probability of a strong eastern Pacific El Niño for the austral summer 2023–2024 is consistent with the LRP explanations and known El Niño predictors. On the other hand, the neutral values for *C* should be taken with care, considering the underestimation of the prediction for 2015–2016, although the unfavorable Atlantic influence appears to also be consistent with a limited El Niño strength.

In this study we did not take into consideration any information posterior to May 2023, not even implicitly, to avoid unintentional data leakage. However, in the interest of the readers, we have included but not discuss the IGP-UHM AI model’s forecast at the time of publication (Supplementary Fig. 2), i.e., with June–July–August initial conditions.

## Discussion

In this study, we developed a new prediction system of El Niño-Southern Oscillation diversity, focused on predicting the E and C indices for eastern and central Pacific sea surface temperature variability, specifically trained on the occurrence of strong El Niño events in the eastern Pacific. The prediction system consists of the IGP-UHM AI deep learning model v1.0, based on a convolutional neural network (CNN), and explainable artificial intelligence (XAI) diagnostics based on the layerwise relevance propagation (LRP) method. The inclusion of XAI diagnostics in the system allows its evaluation by human forecasters for consideration in operational assessments. To enhance the operability of the model, we use four real-time observable predictors (SST, SSH, zonal and meridional wind), in contrast to prior studies that use SST and heat content.

We present a 12-month forecast initialized with data from March–May 2023, which indicates the persistence of warm El Niño conditions, but with decreasing strength, into the 2023–2024 austral summer, similar to 2015–2016 and much weaker than 1997–1998. The comparison of the LRP explanations for 2023 with the other cases provides an objective basis for assessing the credibility of the forecast, particularly pointing not only to the absence of the atmospheric-ocean coupling in the equatorial Pacific and of favorable northerly wind anomalies in the eastern Pacific but also to the negative influence of warm anomalies in the tropical Atlantic.

The fact that the IGP-UHM AI model was specifically trained for strong eastern Pacific El Niño events and with data up to 1984 limits its capabilities for predicting central Pacific warming, such as during 2015–2016. For operational purposes, the model will be fine-tuned using all of the data available and, since every event offers new information on ENSO diversity and climate change, the updating of the model should be done yearly.

In addition to case studies of successful predictions (1997–1998 and 2015–2016), we also highlighted a false positive case, to illustrate the need for assessment by human forecasters of the credibility of the model’s predictions, which can be guided by XAI diagnostics.

Ultimately, the question still remains, what are the limits of ENSO predictability, and whether our current models (dynamical, statistical, AI) can reach those limits? Our results indicate that ENSO nonlinearity is a constraint for model skill, which agrees with prior studies that have shown that periods of stronger ENSO variance are more predictable^50^. This means that if greenhouse gas forcing leads to an increased frequency of strong eastern Pacific El Nino events^23^, models may be able to make improved predictions of their evolution, with significant implications for anticipating and mitigating their impacts.

## Methods

### CMIP models and reanalysis data

The IGP-UHM AI model uses as input sea surface temperature (SST), sea surface height (SSH) as a proxy for the heat content, and zonal (U) and meridional (V) wind output from the historical (1860–2014) simulations with 21 coupled General Circulation Models (GCMs) participating in the Coupled Model Intercomparison Project Phase 6 (CMIP6). Because our model focuses on the prediction of ENSO diversity indices, we only use the subset of CMIP6 models that better simulate ENSO nonlinearity and diversity as measured by the coefficient $\alpha$ of a quadratic fit in the phase space of the first and second principal component of SST anomalies in the tropical Pacific, proposed by ref^22^. Furthermore, we selected some ensemble members from the historical GCM simulations to create an independent training and development set. By doing so, we aim to tune the IGP-UHM AI model without creating any kind of dependency between the model performance and the training data. Before any training is performed, a second GCM selection is conducted based on the number of strong events in each simulation, leaving out those that don’t have at least 7 strong events. We then use 114 years (1871–1984) of data from the Simple Ocean Data Assimilation^60^ (SODA) to fine-tune the model and correct for model biases. To test the model performance, the Global Ocean Data Assimilation System (GODAS) reanalysis^61^ was used for the period 1990–2022 as we take these values to be our ground truth. We leave a 5-year gap between the training/validation period and the testing period to avoid any impact of oceanic memory, similarly to ref.^37^. Anomalies were computed for all the variables used in this study and were coarsened to a 5$^{\circ } \times 5^{\circ }$ resolution in order to improve model performance by enhancing the spatial signature of major features. Lastly, we removed the low signal trend from the GCM data by using a 5th-order Butterworth filter. The wind data used as input with SODA and GODAS was from NOAA-CIRES 20th Century Reanalysis v2^62^ and NCEP-DOE Reanalysis 2^63^, respectively.

For comparison of the prediction skill of the IGP-UHM AI model with a dynamical model, we use monthly sea surface temperature data from the Canadian Seasonal to Interannual Prediction System version 2.1 (CanSIPSv2) GEM5-NEMO climate forecast model^47^, obtained from the North American Multi-Model Ensemble (NMME)^64^. The data is available for the 1981–2018 hindcast period and the 2016–2021 forecast period.

The four variables in the input data were scaled so that their values are comparable, which also helps make the LRP relevance values more comparable. Specifically, for the CMIP GCM data the SST, SSH, zonal and meridional winds were scaled by 1.28$^\circ$, 7.1 cm, and 1.85 m/s and 1.2 m/s, respectively. These values were taken as the maximum in the spatial distribution of the corresponding standard deviations. In the case of the SODA data, the scalings were 1.36$^\circ$, 13.86 cm, and 1.97 m/s and 1.14 m/s.

### E and C indices

The *E* and *C* indices were computed following ref^2^, i.e., by applying a 45$^\circ$ rotation of the first two principal components of SST anomalies in the equatorial Pacific. For the CMIP6 models, the principal components were obtained from the first ensemble member for each GCM for the 1850–2014 period. For SODA and GODAS, the 1971–2000 and 1991–2020 periods were used, respectively. In the case of the GEM5-NEMO, the indices were obtained from ref^24^, who calculated the principal components from the anomalies, separately for each lead time. Coefficient $\alpha$, the metric for ENSO nonlinearity in models, was calculated in the phase space of the first and second principal component of the December–January–February SST anomalies, as in ref^22^.

### IGP-UHM AI model

#### Architecture

The IGP-UHM AI model is a Convolutional Neural Network (CNN) whose architecture has a similar inner design to the CNN for the NINO3.4 index prediction of ref.^37,38^. The input is composed of 3 consecutive months of 4 anomaly fields (SST, SSH, U, V) that are known to be precursors of El Niño events. These variables are transformed to have unit variance so that the convolutional kernel can be trained ignoring data scales. For the convolutional process, we used a $3\times 4 \times 8$ 3D convolution kernel with a stride of 3 in the first dimension (time); by doing so we force the model to train on each variable independently without mixing different spatial evolution patterns in time at the first convolutional layer. The subsequent convolutional processes use $3\times 2 \times 4$ kernels and behave as 2D convolutions due to the shrinking in the time dimension by the selected combination of filter depth and stride. This configuration allows a 3D (volumetric) convolution on the input features only. Early stopping was used as a regularization method to prevent the model from over-fitting. The model output consists of the *E* and *C* index forecast at 12-month lead times, as well as the input month in terms of harmonics (sine, cosine), which forces the model to learn seasonality. Lastly, the model also outputs a classification label for whether the input initial conditions can lead to the occurrence of a strong eastern Pacific El Niño event in the following January. This output is used in conjunction with the XAI method to obtain explanations for the strong El Niño forecasts. The classification label used for training was prepared by tagging the 12 previous months to the January target season where the *E* value is above the 1.5 threshold. We choose to have multiple outputs connected to the same fully connected layer as we determined that it aided the model’s forecast skill and representation of the evolution of the ENSO indices. In order to enhance the statistical robustness of our results, we trained the same configuration multiple times and obtained an ensemble of 30 versions of the model with the same architecture but different weights by having different random data selections during training.

#### Train-Validate-Test prior to finetuning

Prior to using any observational data to finetune and test the model, we use only GCM data to train, validate, and test the hyperparameters of the model. The GCM data were split into four major groups for training (TRAIN), development/validation (DEV1, DEV2), and testing (TEST) (Table 1). The splits were conducted with respect to each GCM’s ensemble members so that each GCM participates with one ensemble member in the training dataset. This allows us to carefully perform independent evaluations of model skill without incurring data leakage. As discussed above, we subselected 21 GCMs based on their skill in simulating ENSO diversity; each model has approximately 153 years of monthly data, resulting in 1,835 samples per model, and a total of 38,535 training samples for the 21 models. For the two validation datasets (DEV1,DEV2), we randomly selected 7 simulations from the GCMs that provided more than one ensemble run, while for the TEST set we selected 12 simulations from the GCMs that provided at least 3 ensemble runs. There is no overlap between the training, validation, and testing datasets. Furthermore, to avoid bias in the training for non-extreme events due to class imbalance (only 7.7% of training data is classified to be an initial condition that led to an extreme event) we perform random data sampling from both data pools (extreme and non-extreme) to get an even split of extreme vs non-extreme initial conditions. This random sampling is performed at every training step, without repetition, until it runs out of samples from the extreme events data pool, from where it shuffles the data and the sampling begins again.

**Table 1:** Sample size per dataset used for training, validation, and testing of the IGP-UHM AI model using only CMIP6 GCM output, before any fine-tuning that uses observational data is performed.

Process	Set	Sample size
Training	TRAIN	38,535
Validation	DEV1	12,845
Validation	DEV2	12,845
Testing	TEST	22,020

Each sample contains 3 consecutive months.

#### Hyperparameter tuning

The hyperparameter tuning was performed by doing a grid search on the learning rate and batch size space. The number of layers and size of filters for both the convolutional and fully connected processes were kept the same as ref.^38^ as these combinations were shown to yield optimal results. As the model was early-stopped when it performed the best with the DEV1 set, the evaluation of skill during training (not shown) was made using the DEV2 set to prevent data leakage. This allowed us to assess the IGP-UHM AI skill by evaluating a set of metrics (CSI and correlations) with an independent set of GCM simulations. Further analysis of the model performance, before any fine-tuning, was conducted with the reserved TEST set. The number of epochs for fine-tuning was carefully selected to avoid overfitting to the reanalysis data.

#### Prediction skill metrics

We considered two skill metrics: The first is the correlation coefficient between the monthly observed and predicted *E* and *C*, calculated for each initial condition month and for all lead times. This was calculated for the IGP-UHM AI model, the GEM5-NEMO model, and a hypothetical AR1 model (i.e., the lagged autocorrelation).

The second metric is the Critical Success Index (CSI) for the categorical forecast of the occurrence of a strong eastern Pacific El Niño ($E>1.5$) in January. The aggregate CSI is obtained by pooling the January forecasts with initial conditions from the preceding 12 months. We also calculate the May CSI, which is based on the January forecast with initial conditions from the preceding May. The CSI is adequate for rare events and is calculated as the number of true positive (TP) forecasts divided by the sum of the number of true positive, false positive (FP), and false negative (FN) forecasts, as shown in Eq. (1).

1 $$\begin{aligned} CSI = \frac{TP}{TP + FP + FN} \end{aligned}$$

For the correlations, we considered the mean *E* and *C* from our 30-model ensemble. In the case of the CSI, a positive result is defined when the average probability of the ensemble classification output exceeds 50%.

### Layerwise Relevance Propagation

Layerwise Relevance Propagation (LRP)^65^ is an explainable AI method used to gain insight from the CNN output by assigning a relevance score to the input variables. We use this method to explain the decision-making of the model on the classification output, specifically on the neuron that classifies whether the initial conditions are a precursor of a strong eastern Pacific El Niño event, such that the sum of the LRP relevance values corresponding to each of the input values equals the output of this neuron. We used the composite version of LRP^66^, which yields the most consistent results among the different versions^36^. The relevance score highlights spatial features that strengthen or weaken the model classification score. We focused the LRP application on the classification output since the application of XAI techniques to regression problems (here, the *E* and *C* output) is less mature^67^.

### Supplementary Information

Supplementary Figures.

## Data Availability

All CMIP6 data are publicly available through the Earth System Grid Federation datanodes at https://esgf-node.llnl.gov/projects/esgf-llnl/. All observational and reanalysis datasets used in the current study are available from the cited authoritative sites.

## Code availability

The architecture and weights of the IGP-UHM AI model v1.0 are published at https://huggingface.co/GRiveraTello/IGP-UHM-v1.0.
