# Supplementary material for: Explained predictions of strong eastern Pacific El Niño events using deep learning
Source: Sci Rep. 2023 Nov 30;13:21150. doi: 10.1038/s41598-023-45739-3 (PMC10689815; doi:10.1038/s41598-023-45739-3)
Supplement: Supplementary file 1 — Supplementary Figures. [file 41598_2023_45739_MOESM1_ESM.pdf]

# Supplementary Information for:

## Explained predictions of strong eastern Pacific El Niño events using deep learning

Gerardo A. Rivera Tello[1,2*]
& Ken Takahashi[1]
& Christina Karamperidou[2]

[1]Instituto Geofísico del Perú, Lima, Perú

[2]Department of Atmospheric Sciences, School of Ocean and Earth Science and Technology
University of Hawaiʻi at Mānoa, Honolulu, HI, USA

[*]Correspondence to: griverat@hawaii.edu

**This PDF file includes:**

- Supplementary Figures 1 – 2

# Supplementary Figures

- **Supplementary Figure 1:** IGP-UHM model predictions of E and C index with May initial conditions for the observational testing period (1990-2022).
- **Supplementary Figure 2:** IGP-UHM AI model predictions and explanations for Jan 2024 with August initial conditions.

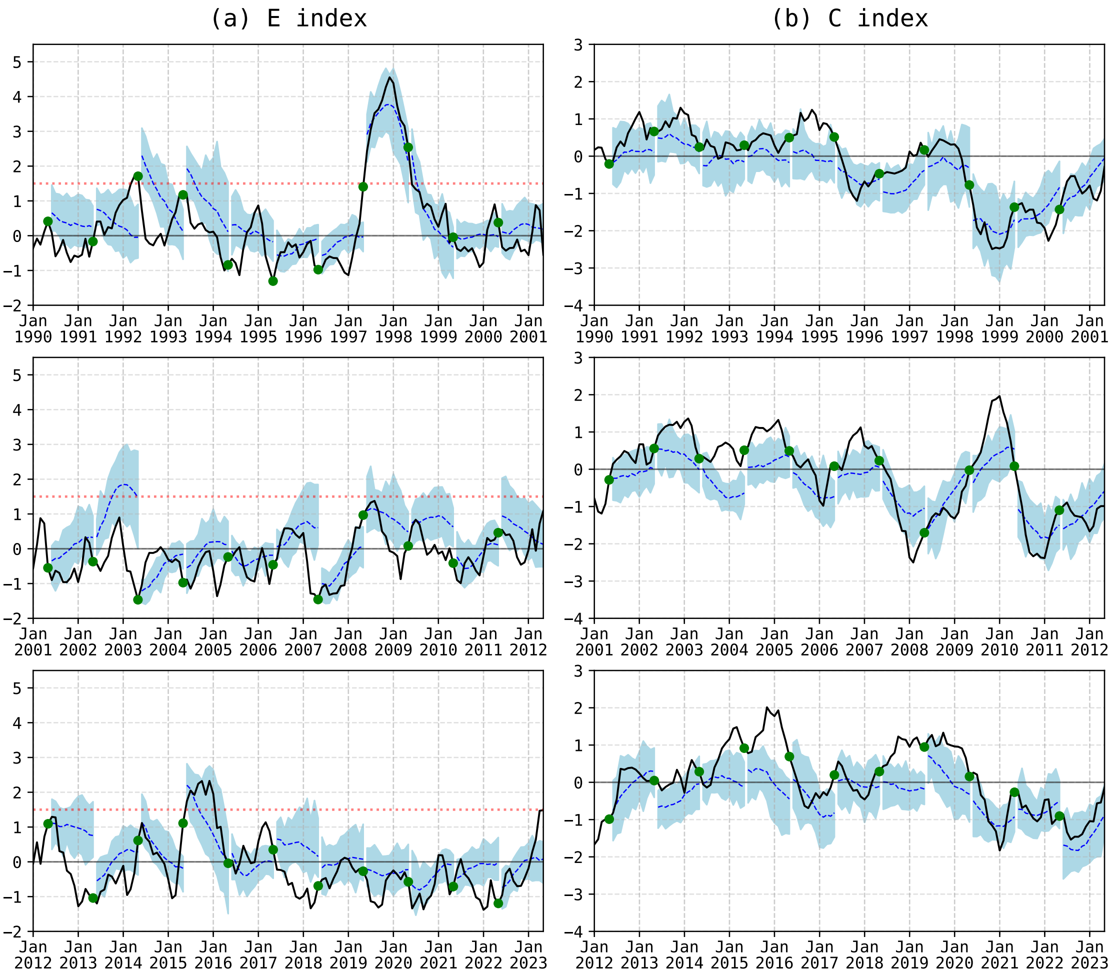


**Figure 1:** IGP-UHM model predictions of (a) E and (b) C index with May initial conditions for the observational testing period (1990-2022). The ensemble mean is shown in the blue dashed line, and the ensemble spread in light blue shading. Green dots indicate May conditions.

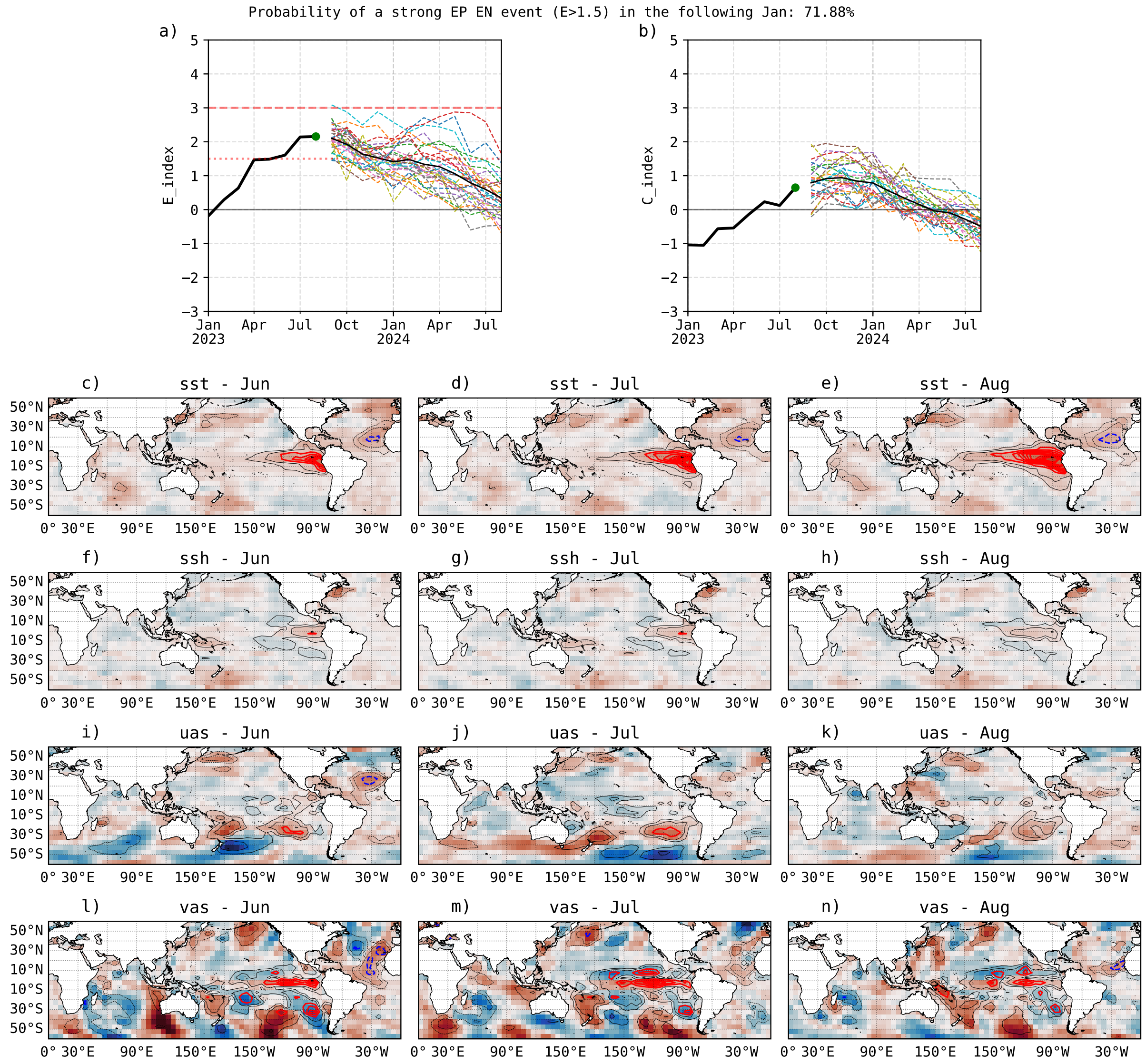


**Figure 2:** IGP-UHM AI model forecasts of a) $E$ and b) $C$ with August 2023 initial conditions, with observed values in black. c-n) Predictor (shading) and LRP relevance (contours) maps for c,f,i,l) June, d,g,j,m) July, and e,h,k,n) August 2023. The LRP maps indicate the conditions that contribute positively (solid contours) and negatively (dashed contours) to the January 2024 prediction.
